# Biogas Production from Physicochemically Pretreated Grass Lawn Waste: Comparison of Different Process Schemes

**DOI:** 10.3390/molecules25020296

**Published:** 2020-01-11

**Authors:** Georgia Antonopoulou, Dimitrios Vayenas, Gerasimos Lyberatos

**Affiliations:** 1 Institute of Chemical Engineering Sciences, Stadiou, Platani, GR 26504 Patras, Greece; dvagenas@chemeng.upatras.gr (D.V.); lyberatos@chemeng.ntua.gr (G.L.); 2 Department of Chemical Engineering, University of Patras, GR 26500 Patras, Greece; 3 School of Chemical Engineering, National Technical University of Athens, GR 15780 Athens, Greece

**Keywords:** grass lawn waste, anaerobic digestion, biochemical methane potential, pretreatment, whole slurry, separated fractions, alkali, acid, energy balance, economical assessment

## Abstract

Various pretreatment methods, such as thermal, alkaline and acid, were applied on grass lawn (GL) waste and the effect of each pretreatment method on the Biochemical Methane Potential was evaluated for two options, namely using the whole slurry resulting from pretreatment or the separate solid and liquid fractions obtained. In addition, the effect of each pretreatment on carbohydrate solubilization and lignocellulossic content fractionation (to cellulose, hemicellulose, lignin) was also evaluated. The experimental results showed that the methane yield was enhanced with alkaline pretreatment and, the higher the NaOH concentration (20 g/100 gTotal Solids (TS)), the higher was the methane yield observed (427.07 L CH_4_/kg Volatile Solids (VS), which was almost 25.7% higher than the BMP of the untreated GL). Comparing the BMP obtained under the two options, i.e., that of the whole pretreatment slurry with the sum of the BMPs of both fractions, it was found that direct anaerobic digestion without separation of the pretreated biomass was favored, in almost all cases. A preliminary energy balance and economic assessment indicated that the process could be sustainable, leading to a positive net heat energy only when using a more concentrated pretreated slurry (i.e., 20% organic loading), or when applying NaOH pretreatment at a lower chemical loading.

## 1. Introduction

Anaerobic digestion (AD) is a mature and well-established technology worldwide for producing bioenergy in the form of methane. In order to improve its efficiency and reduce the process cost, many efforts have been made on finding alternative feedstocks, based on their availability and renewability [1]. Grass lawn (GL) waste coming from gardening or cuttings of sports fields, is nowadays an abundant carbon source, accounting for a significant fraction of organic municipal solid waste (biowaste) [2,3] and is generated even on a daily basis throughout the developed countries. Currently, this waste stream is usually burned, discarded or disposed in landfills [4], depending on the solid waste management strategy, which is followed by a specific country, causing environmental problems.

However, this type of waste is a valuable source of bioenergy, due to its high organic- and specifically carbohydrate-content [5], which remains unexploitable. Thus the possibility of using GL waste as alternative biomass feedstock for AD is quite appealing. Despite its potential use, the complex lignocellulosic structure of GL waste, limits the accessibility of microorganisms and enzymes, restricting thus its digestion. For this reason, an appropriate pretreatment method should be applied in order to remove the structural and compositional barriers and to improve the AD yields [1]. For GL waste, methods including chemical (through alkali or acid addition), physical (e.g., ultrasound, microwave, ionizing radiation), biological (e.g., enzyme and bacteria) or combined processes have been proposed so far, as pretreatment to enhance bioconversions, towards mainly biohydrogen production [5,6,7]. Up to now, limited studies have reported methods for enhancing the AD of GL waste. Tsapekos et al. [8] applied different mechanical pretreatment methods on ensiled meadow grass, or meadow grass without ensiling [9] to investigate their effect on biomass biodegradability and biochemical methane potential (BMP). Khor et al. [10] investigated the possibility of combining extrusion and Ca(OH)_2_ pretreatment to improve storability, availability for biodegradation after storage and BMP of ensiled grass, while Yu et al. [11] investigated the effects of different pretreatments, including ozone, soaking aqueous ammonia (SAA), combined ozone and SAA and size reduction to enhance volatile fatty acid (VFA) and bio-methane production when GL was used as substrate. Finally, Antonopoulou et al. [12] applied SAA to enhance biodegradability and BMP of grass lawn, under different organic loadings.

The aim of the present study was to compare the effects of several pretreatment schemes on the structural and compositional characteristics of GL waste, as well as on the BMP under different process schemes. Specifically, acid and alkali pretreatment methods of GL waste were carried out and compared for the first time. In particular, three different inorganic acids (H_2_SO_4_, H_3_PO_4_ and HCl) and an alkali (NaOH) were tested at three different concentrations (2–20 g/100 gTS) and the impact of each pretreatment was assessed, in a comparative way, through techniques such as Scanning Electron Microscopy (SEM) and IR spectroscopy. Compositional analysis after all pretreatment methods, was used to assess the effect of different pretreatments on the lignocellulosic fractionation of GL waste.

An additional issue is whether it is preferable use the whole slurry resulting from pretreatment for the production of methane or whether it is worth to separate the solid and liquid fractions obtained from the pretreatment and use them separately for methane production. Use of the whole slurry instead of the separate fractions has the advantage of utilizing all the sugars of the pretreated slurry. In addition it leads to reduced process costs, since the step of separation and that of detoxification (to remove the inhibitors from the hydrolysate) are not needed [13].

Based on the individual pretreatment and BMP experiments, an integrated process for AD of GL waste is proposed, contributing to the reduction of a significant fraction of biowastes, while simultaneously producing energy in the form of methane.

## 2. Results and Discussion

### 2.1. Chemical Composition and Structure of GL before and after Pretreatment

The composition of GL waste used in this study was: total solids (TS) (%) = 92.2 ± 0.1, volatile solids (VS) (g/100 gTS) = 83.4 ± 0.1, cellulose (g/100 gTS) = 20.4 ± 0.1, hemicellulose (g/100 gTS) = 24.0 ± 2.0, lignin (g/100 gTS) = 12.3. ± 1.2, extractives (g/100 gTS) = 25.6 ± 3.1 and proteins (g/100 gTS) = 10.5 ± 0.5. Compared to the compositional analysis reported by other studies, the holocellulose content seems to be similar, while the lignin content is lower than that reported (20.39%. reported by Yu et al. [11]).

**Figure 1:**
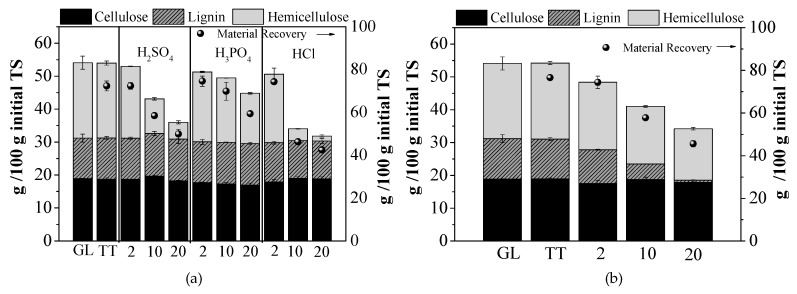
The effect of pretreatment on solid material recovery (right anex), as well as on the fractionation of grass lawn (GL) waste, in terms of cellulose, hemicellulose and lignin, during (**a**) thermal treatment (TT) at 120 °C for 1 h and acid (H_2_SO_4_, H_3_PO_4_, HCl) pretreatment, at the concentrations of 2, 10 and 20 g/100 gTS; (**b**) TT at 80 °C for 24 h and alkali (NaOH) pretreatment at the concentrations of 2, 10 and 20 g/100 gTS, respectively.

Figure 1a,b summarise the effect of different pretreatments (Figure 1a the acidic and Figure 1b the alkali ones) on the fractionation of biomass in terms of lignin, cellulose and hemicellulose. These values are expressed per kg of initial TS, taking into account the solid material recovery of biomass, which is presented in the right axis of both figures. It should be mentioned that during all types of pretreatment, the material recovery associated with the mass loss during pretreatment was less than 100% and the loss of biomass increased with pretreatment severity, leading to a higher material solubilisation. For example, the percentage material recovery (100 − loss of biomass (%)) after treatment with 2 g H_2_SO_4_/100 gTS was 72.4% and after treatment with 20 g H_2_SO_4_/100 gTS, it decreased to 49.9%. A material recovery ranging 64.1 and 94.5%, depending on the pretreatment conditions applied, was obtained by Antonopoulou et al. [14] who tested different acid and alkali pretreatment methods on sunflower straw biomass.

Acid pretreatment resulted in reduction of the hemicellulose fraction (due to its solubilization), and the removal of hemicellulose increased with the acid concentration (Figure 1a). Thus, pretreatment with 10 or 20 g HCl/100 gTS caused a reduction of hemicellulose by 84.37 and 93.4%, respectively, while when using H_2_SO_4_ or H_3_PO_4_ at the higher concentration of 20 g/100 gTS, the hemicellulose fraction was reduced by 77.9 and 33.8%, respectively. The acid pretreatment was not effective in removing cellulose and lignin, as confirmed also by other studies [1,13] reporting that under acidic conditions the main reaction that occurs is the hydrolysis of hemicellulose, especially xylan, while lignin is hardly solubilized, but is disrupted to a high degree, increasing cellulose susceptibility to enzymes.

As it can be seen from Figure 1b, alkali pretreatment was more effective in lignin breakdown, causing depolymerization and cleavage of lignin-carbohydrate linkages. The higher the NaOH concentration used, the higher was the lignin degradation observed. Specifically, when 2, 10 and 20 g NaOH/100 gTS were applied, a lignin removal of 16.7, 61.7 and 94.5%, respectively, was observed, indicating the effectiveness of the method for lignin decomposition. Under the same conditions, the hemicellulose removal efficiency was 10.3, 23.5 and 31.8% for 2, 10 and 20 g NaOH/100 gTS, respectively, while cellulose was not influenced at all. The fact that alkali pretreatment methods (such as soaking in NaOH or NH_3_ based aquatic solutions), have been shown to be efficient in lignin removal, while the preservation of mainly cellulose has also been confirmed by other studies [1,12,15,16].

A t-Test of the lignocellulosic fractionation of GL waste, before and after H_2_SO_4_ pretreatment, showed that the average lignin and cellulose contained in GL were not affected significantly. For treatment with H_2_SO_4_ at all concentrations, the hemicellulose fraction before pretreatment was significantly higher than the respective fractions after pretreatment (*p* = 0.0008, *p* = 0.0003 and *p* = 0.003 < 0.05 for 2, 10 and 20 g/100 gTS, respectively). The same trend was observed for treatment with HCl and H_3_PO_4_. Regarding alkaline pretreatment, NaOH at all concentrations led to similar results, where statistical difference was found between hemicellulose and lignin, before and after pretreatment.

**Figure 2:**
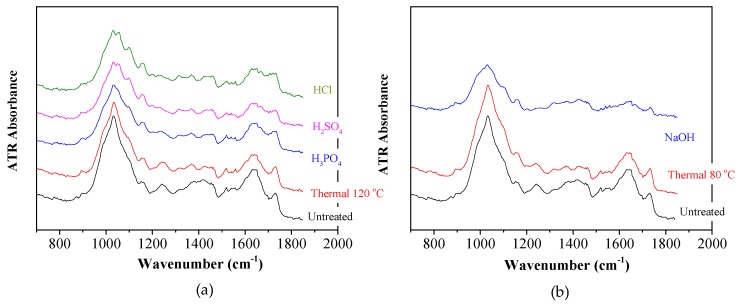
Attenuated Total Reflection (ATR) spectra of raw and (**a**) thermal treatment (TT) at 120 °C for 1 h and acid (H_2_SO_4_, H_3_PO_4_, HCl) pretreatment, at the concentration of 20 g/100 gTS; (**b**) TT at 80 °C for 24 h and alkali (NaOH) pretreatment at the concentration of 20 g/100 gTS, respectively.

In Figure 2, representative Attenuated Total Reflection (ATR) spectra of raw, acid (H_2_SO_4_, H_3_PO_4_, HCl) at the higer concentration of 20 g/100 gTS and thermally pretreated, at 120 °C, GL waste, (Figure 2a), as well as the respective of thermally (80 °C) and alkaline pretreated (NaOH, 20 g/100 gTS (Figure 2b)) GL waste, are presented in a spectral range of 600 to 1800 cm^−1^, in order to verify the chemical changes of the lignocellulosic material before and after pretreatment. The pretreated samples exhibited increased intensities in the regions of 1000–1200 cm^−1^ and 1500–1700 cm^−1^.

The most important absorption bands commonly found in lignocellulosic biomass samples are observed at 894, 1043, 1242–1256, 1518, 1640 and 1730 cm^−1^ [17] and are associated with the three major lignocellulosic components. Fundamentally, cellulose is formed by glycosidic linkages and hydroxyl groups with a small amount of carboxyl, while hemicellulose and lignin are predominated by ether bond, with hemicellulose also characterized by a significant amount of carboxyl groups [18]. As shown in Figure 2a,b, the spectra of untreated and thermally treated GL waste are similar, indicating that thermal treatment without chemical agent addition did not significantly influence the lignocellulosic fraction, which was also confirmed by Figure 1a,b. The band at 894 cm^−1^ corresponding to C-H deformation/C-O-C stretching at β-1,4 glycosidic linkages, due to the amorphous part of cellulose, is intense in chemically pretreated samples, indicating the decrease in crystalline to amorphous fraction of cellulose, due to the different pretreatment methods. The signal of C-O, C-C and C-OH stretching vibrations at 1043 cm^−1^, related to cellulose, hemicellulose and lignin [19] in the spectra of the chemically pretreated samples, corresponded to different peaks, compared to the thermally treated or raw GL waste, respectively. Moreover, the C-O vibrations of G rings of lignin at 1242–1256 cm^−1^, the aromatic skeletal vibration of C=C bond of lignin at 1518 cm^−1^ [20] and the C=O stretching vibration in carbonyl of lignin [17] were represented by lower or no peaks, in the spectrum of alkaline treated sample, compared to the untreated one, as shown in Figure 2b. This could be attributed to the high reduction of lignin (94.5%) which took place due to alkaline pretreatment, as also confirmed by the characterization of the lignocellulosics (Figure 1b). Finally, the signal of ester bond due to C=O stretching in unconjugated ketone, carbonyl and ester groups related to xylan [21] is less intense in the acid treated samples, due to the high solubilization of hemicellulose, which took place under these pretreatment conditions.

**Figure 3:**
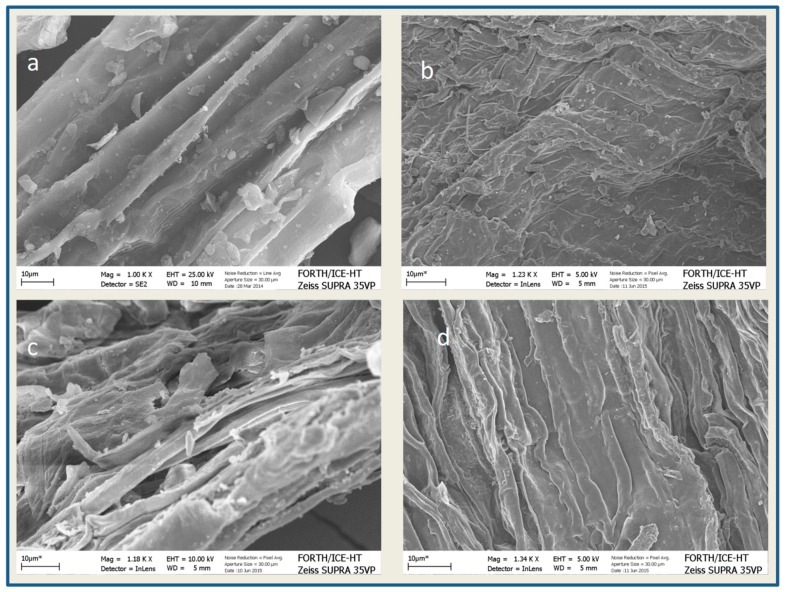
SEM images of raw (**a**), alkali (NaOH) (**b**) acid (H_2_SO_4_) (**c**) and acid (HCl) (**d**) pretreatment, at the concentration of 20 g/100 gTS, respectively.

In Figure 3, representative SEM images of: (a) raw, (b) alkali (c) acid with H_2_SO_4_ and (d) acid with HCl at 20 g/100 gTS, are presented. It is obvious that there is a different morphology after different pretreatment methods, compared to the raw sample. Specifically, treatment with 20 g H_2_SO_4_/100 gTS led to a different surface with disrupted parts, also containing pinholes and gaps as well as parts with a smoother outer layer. The smoother surface is evident in Figure 3b,d too, where it is obvious that HCl and NaOH pretreatment methods led to a different surface structure compared to the untreated GL waste. Similar images were also obtained from Yang and Wang [5] when using HCl (1% *w*/*w*) for 30 min at 100 °C as a pretreatment method of grass to enhance fermentative hydrogen production. Antonopoulou et al. [12] observed also a smoother surface of GL when applying SAA for 3 days at 22 °C, as a pretreatment method to enhance the BMP of this substrate. Also, Kang et al. [16] observed a deconstructed and more accessible surface after pretreatment of *Pennisetum hybrid,* a perennial grass with 2% NaOH at 35 °C for 24 h in order to enhance its anaerobic digestibility.

### 2.2. BMP of GL Waste before and after Pretreatment

#### 2.2.1. BMP of Untreated GL

The calculated methane production, after subtraction of the methane produced from blank experiments was 62.44 ± 0.36 mL, corresponding to a BMP of 260.65 ± 0.04 L CH_4_/kg GL waste or 282.60 ± 0.04 L CH_4_/kg TS or 339.86 ± 0.05 L CH_4_/kg VS. The BMP of the GL waste used in the present study was comparable with that of ensiled meadow grass (372 ± 52 L CH_4_/kg) [8] and GL (402.5 L CH_4_/kg) [11], but higher than that of giant reed [22] or common reed [23] (188 L CH_4_/kg). In any case, the BMP of the untreated GL waste is higher than that of other lignocellulosic feedstocks and this could be attributed to the low lignin content, since the latter is negatively correlated to the BMP [24]. Apart from the low lignin content, GL waste has high holocellulose (sum of cellulose and hemicellulose) and high extractives, content, which are possible sources for high methane productions.

#### 2.2.2. BMP of the Whole Pretreatment Slurry

**Figure 4:**
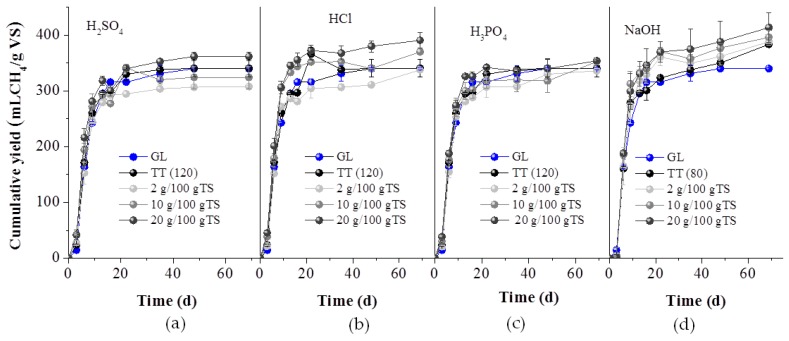
Cumulative methane yield of untreated (GL), thermally treated at 120 °C for 1h (TT (120)) or 80 °C for 24 h (TT (80)) and acid, with H_2_SO_4_ (**a**), HCl (**b**), H_3_PO_4_ (**c**) and alkali with NaOH (**d**), at the concentrations of 2, 10 and 20 g/100 gTS, respectively.

BMP experiments were conducted for the untreated GL waste, the whole pretreatment slurry (mixture of liquid and solids obtained after all pretreatment methods) as well as the separate fractions i.e., at the liquid (hydrolysate) and the solid fractions obtained after acid and alkali pretreatment methods used. In Figure 4, the effect of all pretreatment methods on the BMP of the whole slurry is presented, expressed as mL methane per g of initial VS. It is obvious that the kinetics of the process were not enhanced by the pretreatment, since more than 80–90% of the total biomethane of all experiments (even of the control- with untreated GL waste) was produced within about 13 days. It is also obvious that all alkali pretreatment methods affected positively the methane yield and the higher the NaOH concentration, the higher was the methane yield. Thus, treatment with 2, 10 and 20 g NaOH/100 gTS led to 389.0 ± 7.0, 397.7± 12.2 and 414.8 ± 26.5 L CH_4_/kg VS, respectively, corresponded to a 14.74, 17.01 and 22.05% BMP increase, compared to the BMP of untreated GL waste. The increase of BMP can be attributed to the lignin reduction which occurred under alkaline pretreatment (Figure 1b) and was more intense at the higher NaOH concentration. During alkaline pretreatment, a saponification and cleavage of lignin- carbohydrate linkages has been reported to occur [25] rendering the structure smoother and increasing thus the biodegradability during the AD.

In the literature, application of an alkali (through the use of NaOH, Ca(OH)_2_ or ammonia solution) for enhancing AD and/or the BMP of a lignocellulosic substrate, is commonly reported [1,26]. For instance, Jiang et al. [22] observed an improved enzymatic digestibility and biogas production from giant reed, after its treatment with NaOH and reusing the pretreatment leachate. Yu et al. [11] enhanced the specific methane yield of GL from 402.5 mL CH_4_/gVS (untreated) to 481 mL CH_4_/gVS, after treatment with SAA, while Kang et al. [16] improved the methane yield of *Pennisetum Hybrid* by 21%, after treatment with 2% NaOH (35 °C, 24 h).

Acid pretreatment enhanced slightly the BMP, i.e., a 4% increase was achieved through the addition of 20 g H_3_PO_4_/100 gTS, 6.9% due to 20 g H_2_SO_4_/100 gTS and 15.8% due to 20 g HCl/100 gTS. Recent studies report that acid pretreatments are appropriate for fermentative processes, such as biohydrogen or bioethanol production, due to the solubilization of hemicellulose and not for AD, since they have no effect on lignin [4,14].

#### 2.2.3. BMP of the Solid and Liquid Fractions Obtained after Pretreatment

**Figure 5:**
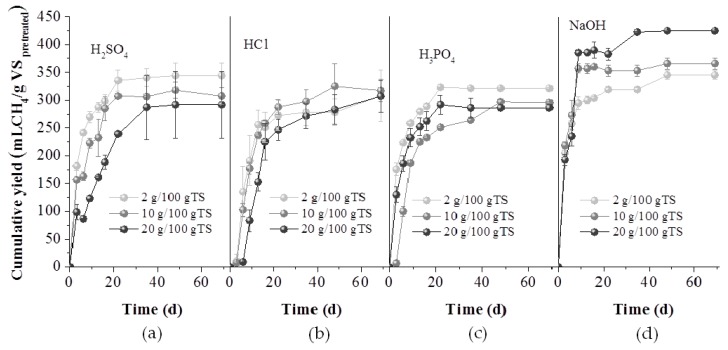
Cumulative methane yield of the solid fraction obtained after pretreatment of GL waste with H_2_SO_4_ (**a**), HCl (**b**), H_3_PO_4_ (**c**) and NaOH (**d**), at the concentrations of 2, 10 and 20 g/100 gTS, respectively.

In Figure 5, the effect of pretreatment on the BMP of the solid fractions obtained after pretreatment is presented, expressed as mL methane per g vs. of the pretreated biomass. It is obvious that for all acid pretreatment methods, the lower chemical concentration of 2 g/100 gTS led to higher methane yields, which could be attributed to the higher holocellulose content in the solid fraction, available for biodegradation from the anaerobic sludge. Since treatment with 10 or 20 g acids/100 gTS led to high hemicellulose removal efficiency, which degraded towards xylose or arabinose and released in the hydrolysate, the biodegradable solid fraction was reduced.

NaOH pretreatment, on the other hand, enhanced significantly the BMP and the higher the NaOH concentration, the higher was the methane yield produced (345.1 ± 7.6, 365.9 ± 9.9 and 424.8 ± 1.5 L CH_4_/kg VS_pretreated_, for 2,10, and 20 g NaOH/100 g TS, respectively). This could be justified by the higher lignin removal that occurred under more severe conditions (higher NaOH concentration). Moreover, holocellulose was not affected significantly by alkali pretreatment and based on Figure 3b it can be concluded that only the surface structure was changed. This fact implies that an increase of the cellulose surface area, which was available for enzymatic attack in the subsequent digestion step, occurred, which is also consistent with other studies [12,13]. Apart from the methane yield, NaOH pretreatment accelerated the kinetics of the process, since more than half of the ultimate methane yield was produced within the first 3 days for all NaOH concentrations.

**Figure 6:**
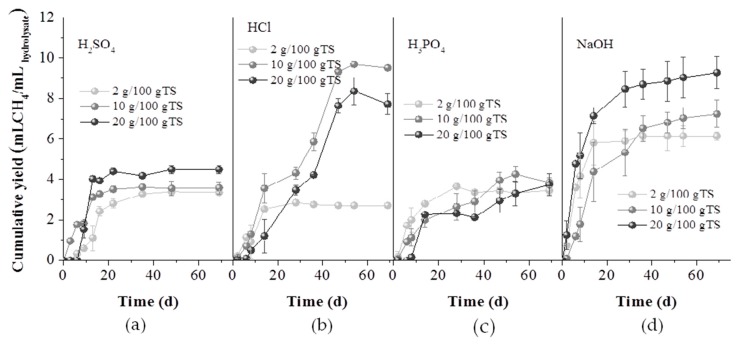
Cumulative methane yield of the hydrolysates obtained from GL waste pretreated with H_2_SO_4_ (**a**), HCl (**b**), H_3_PO_4_ (**c**) and NaOH (**d**), at the concentrations of 2, 10 and 20 g/100 gTS, respectively.

In Figure 6, the BMP of the hydrolysates, expressed as mL methane per mL of hydrolysate, is presented. Contrary to the results from the experiments with the solid fractions, for NaOH and H_2_SO_4_, the higher the chemical concentration of the pretreatment agent, the higher were the methane yields obtained. Pretreatment with 10 or 20 g HCl/100 gTS and 20 gNaOH/100 gTS led to 9.73 ± 0.03, 8.09 ± 0.47 and 9.40 ± 0.97mL/mL_hydrolysate_, respectively. These high values of the BMP could be attributed to the high organic and sugars content of the hydrolysates, which were released during hemicellulose solubilization, as also confirmed by the values of Table 1, in which the main sugar monomers (glucose, xylose and arabinose) as well as the concentration of soluble sugars, are presented.

#### 2.2.4. Comparison of the Methane Yields Obtained from Different Processes

The selection of an overall process scheme is crucial for the process economics. In Table 2, the methane yields of all fractions (whole biomass, solid and liquid fractions) obtained using all pretreatment methods, are presented. For comparison, the yields of the separate fractions have also been expressed in terms of mL CH_4_/g VS_initial_, taking into account the solid material recovery (loss of weight) due to pretreatment. Thus, for the solid fraction, the methane yield was calculated as:

(1) $${\mathrm{CH}}_{4}\;\mathrm{yield}({\frac{L}{\mathrm{kg}\;\mathrm{VS}}}_{\mathrm{initial}})\;=\;{\mathrm{CH}}_{4}\;\mathrm{yield}\left(\frac{L}{{\mathrm{kg}\;\mathrm{VS}}_{\mathrm{pretreated}}}\right)\;\times \;\mathrm{Material}\;\mathrm{Recovery}(\frac{{\mathrm{kgVS}}_{\mathrm{pretreated}}}{{\mathrm{kgVS}}_{\mathrm{initial}}})$$

while for calculating the methane yield of the hydrolysates in terms of mL CH_4_/g VS_initial_, the fact that 100 mL of water were mixed with 5 g TS _initial_ was taken into account, assuming also that no liquid was lost during separation.

Comparing the BMP of the sum of both fractions, expressed in L/kg VS_initial_ with the respective of the whole slurry at each pretreatment method, it is obvious that direct AD without separation of the pretreated biomass was favored in almost all cases. Only treatment with 20 gNaOH/100 gTS led to 427.07 L CH_4_/kg VS, after separation, which corresponded to 25.7% enhancement of the BMP of the untreated GL waste. This value was only 3.3% higher than the BMP of the whole pretreated slurry (413.5 L CH_4_/kg VS), under the same pretreatment conditions. However, the approach of using the whole slurry has the advantage of reduced process costs, since the step of separation is not needed [27]. Thus, taking into account these aspects, treatment with 20 g NaOH/100 gTS and direct AD of the whole slurry, seems to be the most promising scheme.

### 2.3. Energy and Cost Analysis

The experimental results showed that pretreatment with 20 g NaOH/100 gTS for 1d at 80 °C and direct AD of the whole slurry (NaOH-CH_4_), led to higher methane yield, of 413.5 L/kg vs. or 346 L CH_4_/kg TS. In the present study, a preliminary energy balance and economic assessment of the application of alkaline pretreatment was carried out, by comparing the extra cost (i.e., heating and chemical reagent) required for the pretreatment, with the extra energy in the form of methane due to pretreatment [28]. Thus, apart from the scenario in which alkali pretreatment was employed, the scenario of direct AD of GL waste, without pretreatment (CH_4_ of GL) was also analyzed, for comparison reasons.

For realizing the process in full scale, a shredder for milling the grass to the proper size, a tank where the alkaline pretreatment will be carried out, an anaerobic digester for producing biogas from the whole slurry and a combined heat and power (CHP) unit, for biogas exploitation, should be involved.

The energy produced, as estimated by the BMP was found to be 3460 kWh/t TS for the alkali pretreated biomass and 2826 kWh/t TS, for the AD of untreated GL waste (taking into account the energy from methane as 10 kWh/m^3^). From the energy yield, the surplus energy (thermal and electrical) produced by the CHP unit, was estimated and presented in Table 3, and compared with the energy demands of the whole process, such as heat energy requirement (HER), electrical energy for mixing the pretreatment tank and the cost of NaOH. Regarding the CHP generator, it was assumed that a typical unit produced 35% electricity and 50% heat (thermal efficiency).

Regarding thermal energy gain, the surplus heat was calculated as the difference between the heat produced by the NaOH-CH_4_ process and the process without alkaline pretreatment (CH_4_ of GL) (317 kWh/t TS).

In the present study, a solids loading of 5% *w*/*v*, or 50 g TS/L was assumed. However, several researchers assessed the possibility of applying pretreatment methods at higher solid loading (>15% solids, *w*/*w*) [29,30]. Especially, in full scale AD the use of more concentrated slurries (e.g., 20% *w*/*v*) could be feasible. Thus, in the present analysis two different scenario were evaluated: the first scenario in which 50 g TS/L were used (as indicated from the experiments) and the second one, in which a more concentrated whole slurry of 200 g/L (20% *w*/*v*) was used (20g TS per 100 mL of water or aquatic solution of NaOH).

Regarding the pretreatment tank, addition of water or aquatic solution of NaOH should be carried, so as to reach the solids loadings (50 or 200 g TS/L) and a chemical loading of 20 g NaOH/100 g TS. The HER in kWh/t TS, for thermo-alkaline pretreatment (80 °C) of 1 ton TS of GL waste was estimated according to Equation (2) [28]:

(2) $$\mathrm{HER}\;=\;\frac{m\;\times \;Cp\;\times \;\left(T_{final}\;-\;T_{initial}\right)}{3600}$$

where m is the mass of water and substrate in kg; Cp the water specific heat (4.18 kJ/kg °C); T_initial_ and T_final_ in °C is the initial and final temperature of the substrate suspension, assumed as 25 °C; and 80 °C, respectively.

The surplus heat was then compared to the thermal energy requirement for alkaline pretreatment (HER). At a solid loading of 50 gTS/L the surplus heat (317 kWh/t TS) was not sufficient to cover the heat requirement for alkaline pretreatment (1343 kWh/t TS). For the solid loading of 200 gTS/L, the net heat energy (NHE) of alkaline pretreatment was slightly negative (−68 kWh/t TS). Thus, assuming a heat energy recovery from the pretreatment step by almost 80% [31], a positive NHE was achieved for both solid loadings (48.4 and 240 kWh/t TS for solids loadings of 50 and 200 g TS/L, respectively) (Table 4).

Regarding the requirements of electrical energy, only the mixing demands in the pretreatment tank were considered (10.5 kWh/t TS [32]). It should be emphasized that the electricity demands for GL waste grinding and milling were not considered, since these machines were also necessary for the AD of untreated GL. The net electrical energy required for alkaline pretreatment of GL waste at both solids loadings is presented in Table 5.

For the economic assessment, the cost of chemicals from one side and the incomes from the sale to the public grid of electricity surplus (211.4kWh/t TS), from the other, should be compared (Table 5). As assumption an average price for biogas energy in European countries was considered (0.25 €/kWh). From the values of Table 5, it is obvious that the process should be sustainable either by using a lower alkali loading, or by selling with higher price in the public grid.

## 3. Materials and Methods

### 3.1. Biomass Used

GL was collected in the region of Attica, Greece, during gardening. It was initially air dried, then grinded with a house blender (Izzy X3, E560T3, Titanium, Crete, Greece) and milled with a lab grinder (A11 basic, IKA, Staufen, Germany) to powder, passing through a sieve with a pore size of 0.7 mm. Finally, it was air-dried at ambient temperature before being used for the experiments.

### 3.2. Pretreatment Methods Tested

For all pretreatment methods tested, the solids load was 5% *w*/*v*. Acid pretreatment was conducted at 121 °C for 1 h, by the use of three different inorganic acids (H_2_SO_4_, H_3_PO_4_ and HCl) at concentrations of 2, 10 and 20 g/100 g TS, respectively. Alkaline pretreatment was conducted at 80 °C for 24 h, by the use of NaOH at the same concentrations. For comparison, blank experiments, in which only thermal treatment (121 °C for 1 h or 80 °C for 24 h) without any chemical addition, were also carried out. After pretreatment, either the whole pretreatment slurry (liquid and solid fractions obtained after pretreatment) or the two fractions obtained after separation through filtering with 0.7 μm, were used for BMP, in batch reactors. A detailed physicochemical characterisation was also performed in the solid and liquid fractions, as described below.

### 3.3. BMP Experiments

BMP experiments were carried out in duplicate at 35 °C in serum bottles of 160 mL, using a working volume of 100 mL. The experiments were performed according to the modified protocol of Owen and Chynoweth [33] using as inoculum sludge from the anaerobic digester of the Patras wastewater treatment plant, treating municipal sewage sludge and operating at steady state at an hydraulic retention time (HRT) of 15 d. The main characteristics of the sludge were: pH: 8.13, total chemical oxygen demand (T.COD): 25.4 g/L, dissolved COD (d.COD): 0.81 g/L, total suspended solids (TSS): 23.03 g/L and volatile suspended solids (VSS): 14.13 g/L.

BMP tests were performed either at the whole slurry or at the separated fractions, obtained after pretreatment. For the experiments with the whole slurry, 20 mL mixed anaerobic culture, 76 mL water and 4 mL of the whole slurry at a solid loading of 5% *w*/*v*, were used. For the experiments with the solids obtained after pretreatment, 20 mL mixed anaerobic culture, 80 mL water and appropriate amounts of samples were added, in order to acquire the desirable TS content of 2g TS/L. For the experiments with the hydolysates, 20 mL mixed anaerobic culture were seeded with water and appropriate volumes of hydrolysates, so as their final COD concentration, being 2 g/L. For all experiments, the microbial culture was supplemented with 10 mL/L of a (NH_4_)_2_HPO_4_ (7.21 g/L) solution, 10 mL/L of a FeSO_4_·7H_2_O (0.7g/L) solution and 10 mL/L of a trace metals solution [34]. Control experiments for checking the methanogenic biomass activity using glucose and cellulose, as well as blank experiments in order to determine the background gas productivity of the inoculum, were also carried out. The content of the vials was gassed with a mixture of N_2_/CO_2_ (80/20) in order to secure anaerobic conditions. The vials were sealed with butyl rubber stoppers and aluminum crimps and methane production was monitored as a function of time according to Owen and Chynoweth [33].

### 3.4. Analytical Methods

The analytical procedure for samples characterization in terms of their lignocellulosic content is presented in Antonopoulou et al. [14]. Briefly, raw samples were air-dried and then used for ethanol extraction (exhausted extraction for 24 h) [35] prior to the compositional analysis, which was performed according to the National Renewable Energy Laboratory (NREL)’s standard laboratory analytical procedure (LAP) [36]. Detection and quantification of sugar monomers (glucose, xylose and arabinose) were performed with HPLC-RI with an Aminex HPΧ-87H column (BioRad, Marnes-la-Coquette, France) at 60 °C and a Cation H micro-guard cartridge (RioRad) using H_2_SO_4_ 0.006 Ν as an eluent at a flow rate of 0.7 mL/min. For the characterization of the pretreated samples, a separation of liquid and solid fractions was made, through filtering with 0.7 μm filters. The solid fractions were washed with water, air-dried and characterized as described above for the raw samples, but without performing an extraction process prior to the characterization.

The liquid fractions were used for soluble charbohydrates’ content determination, according to Joseffson [37] and for the identification of monomeric sugars (glucose, xylose, arabinoze), using the method described above. The measurements of TS, VS, TSS and VSS as well as of d.COD and T.COD were carried out according to Standard Methods [38]. Raw and extractive-free samples were also used to determine Τotal Kjeldahl Νitrogen (TKN) according to Standard Methods [38] where the crude protein content was estimated by multiplying TKN by a factor of 6.25 [26]. The methane content of the produced biogas was quantified as described in Alexandropoulou et al. [26] while SEM images and IR spectra were obtained as described in Antonopoulou et al. [14].

### 3.5. Statistical Analysis

A two-sample t-test with a threshold *p*-value of 0.05 was applied in order to analyze statistically the effect of pretreatment on the lignocellulosic content of GL waste.

## 4. Conclusions

The experimental results obtained showed that the treatment with acids led to higher hemicellulose solubilization, while lignin removal from the solid matrix was achieved, when grass lawn waste (GL) was treated with NaOH. Higher acids concentrations led to higher solubilization of hemicellulose. The BMP of GL was enhanced with alkaline pretreatment and the higher the NaOH concentration, the higher was the methane yield observed. Comparing the BMP under different process schemes (whole or separated fractions) the experiments indicated that the use of the whole slurry was beneficial for the process yields and economy.

## Figures and Tables

**Table 1:** Concentration of glucose, xylose, arabinose and sugars accompanied by their standard deviations, contained in the liquid fractions obtained after different pretreatment methods.

Pretreatment	Glucose (g/100 gTS)	Xylose (g/100 gTS)	Arabinose (g/100 gTS)	Sugars (g/100 gTS)
Thermal (120 °C)	1.43 ± 0.08	3.98 ± 0.10	-	6.36 ± 0.10
Thermal (80 °C)	1.12 ± 0.04	2.96 ± 0.12	-	5.21 ± 0.18
H_2_SO_4_,2 g/100 gTS	1.12 ± 0.01	3.29 ± 0.08	0.17 ± 0.01	6.71 ± 0.34
H_2_SO_4_, 10 g/100 gTS	2.59 ± 0.15	5.02 ±0.25	3.71 ±0.35	14.77. ± 0.11
H_2_SO_4_, 20 g/100 gTS	3.11 ± 0.03	10.42 ± 2.18	3.56 ± 0.91	15.12 ± 0.14
H_3_PO_4,_ 2 g/100 gTS	1.82 ± 0.04	2.89 ± 0.17	0.52 ± 0.09	6.24 ± 0.03
H_3_PO_4_, 10 g/100 gTS	1.63 ± 0.03	3.07 ± 0.05	1.74 ± 0.06	7.69 ± 0.74
H_3_PO_4_, 20 g/100 gTS	1.73 ± 0.01	3.37 ± 0.02	2.66 ± 0.06	11.17 ± 1.78
HCl, 2 g/100 gTS	1.34 ± 0.01	3.16 ± 0.08	1.70 ± 0.10	8.08 ± 0.12
HCl, 10 g/100 gTS	2.33 ± 0.15	12.64 ± 0.25	4.05 ± 0.07	18.21± 0.69
HCl, 2 20/100 gTS	3.53 ± 0.03	13.28 ± 0.45	4.78 ± 0.23	19.03 ± 0.58
NaOH, 2 g/100 gTS	1.38 ± 0.08	2.12 ± 0.10	n.d.	5.34 ± 0.78
NaOH, 10 g/100 gTS	1.28 ± 0.08	4.52± 0.06	0.46 ± 0.05	6.80 ± 0.20
NaOH, 2 20/100 gTS	1.75 ± 0.05	5.87 ± 0.03	1.99 ± 0.01	8.58 ± 0.30

**Table 2:** BMP of the whole pretreatment slurry and of the separated fractions obtained after all pretreatment methods.

	BMP (L/kg vs. Initial)			
Pretreatment	Whole Biomass	Solid Fraction	Liquid Fraction	Sum
Untreated GL	339.86 ± 1.75		-	
Thermal (120 °C)	340.72 ± 15.83	177.09± 1.84	86.51 ± 2.16	263.6
Thermal (80 °C)	383.70 ± 0.50	211.36± 1.28	105.49± 2.40	316.85
H_2_SO_4_,2 g/100 gTS	307.82 ± 1.62	203.93 ± 3.51	80.51 ± 1.29	284.44
H_2_SO_4_, 10 g/100 gTS	324.25 ± 20.32	174.74 ± 25.56	86.07 ± 7.21	260.81
H_2_SO_4_, 20 g/100 gTS	361.70 ± 7.04	141.18 ± 9.41	107.77 ± 4.93	248.95
H_3_PO_4,_ 2 g/100 gTS	336.19 ± 3.24	211.39 ± 39.5	83.05 ± 4.28	294.44
H_3_PO_4_, 10 g/100 gTS	352.44 ± 4.07	177.62 ± 1.03	92.31 ± 5.50	269.93
H_3_PO_4_, 20 g/100 gTS	354.09 ± 1.062	146.99 ± 0.74	128.33 ± 13.56	275.32
HCl, 2 g/100 gTS	337.50 ± 2.68	222.37 ± 24.2	65.09 ± 1.24	287.46
HCl, 10 g/100 gTS	369.98 ± 6.81	164.72 ± 11.86	233.38 ± 0.83	398.1
HCl, 2 20/100 gTS	390.77 ± 13.92	134.23 ± 6.85	193.99 ± 11.30	328.22
NaOH, 2 g/100 gTS	388.13 ± 5.82	255.68 ± 3.34	147.42 ± 4.80	403.1
NaOH, 10 g/100 gTS	396.31 ± 11.68	214.70± 4.46	173.60± 16.15	388.3
NaOH, 2 20/100 gTS	413.50 ± 26.08	202.06 ± 4.70	225.01 ± 23.20	427.07

**Table 3:** Energy analysis for CH_4_ production from the alkaline pretreated GL (NaOH-CH_4_**)** and for untreated GL waste (CH_4_ of GL).

	NaOH-CH_4_	CH_4_ of GL
Energy from CH_4_ (kWh/t TS)	3460	2826
Thermal energy produced (kWh/t TS)	1730	1413
Electrical energy produced (kWh/t TS)	1211	989.1
Energy produced (heat and electrical from CHP (kWh/t TS)	2941	2402.1

**Table 4:** Energy analysis for NaOH-CH_4_. The solids loadings of 50 and 200 g/L were considered.

Solid Loadings (gTS/L)	50	200
Thermal energy gain (kWh/t TS)^a^	317	317
Heat energy (HE) requirement (kWh/t TS)	1343	385
HE requirement with 80% of heat recovery (kWh/t TS)	268.6	77
Net heat energy (NHE) (kWh/t TS)^b^	−1026	−68
NHE with 80% of heat recovery (kWh/t TS)	48.4	240

^a^ Thermal energy gain corresponds to the difference of heat energies produced by NaOH-CH_4_ minus CH_4_ of GL; ^b^ NHE is the difference between the thermal energy increase and the heat energy requirement for the alkaline pretreatment.

**Table 5:** Energy analysis and economical assessment for NaOH-CH_4_.

	NaOH-CH_4_
Electrical energy	
Electrical energy increase (kWh/t TS)^a^	221.9
Mixing pretreatment tank (kWh/t TS)	10.5
Net electrical energy (kWh/t TS)	211.4
Economic assessment	
NaOH cost (€/t TS)	82.4
Extra net gain (€/t TS),	52.85

^a^ Electrical energy gain corresponds to the difference of electricity energies produced by NaOH-CH_4_ process minus the CH_4_ of GL.
